# Umbilical cord blood level of interleukins used as a predictor of early-onset neonatal sepsis: a comprehensive review

**DOI:** 10.3389/fcimb.2025.1518088

**Published:** 2025-03-18

**Authors:** Maria Andreea Răcean, Maria Oana Săsăran, Cristina Oana Mărginean, Manuela Cucerea

**Affiliations:** ^1^ Department of Pediatrics 4, George Emil Palade University of Medicine, Pharmacy, Science, and Technology of Targu Mures, Târgu Mureş, Romania; ^2^ Doctoral School of Medicine and Pharmacy, George Emil Palade University of Medicine, Pharmacy, Science, and Technology of Targu Mures, Târgu Mureş, Romania; ^3^ Department of Pediatrics 3, George Emil Palade University of Medicine, Pharmacy, Science, and Technology of Targu Mures, Târgu Mureş, Romania; ^4^ Department of Pediatrics 1, George Emil Palade University of Medicine, Pharmacy, Science, and Technology of Targu Mures, Târgu Mureş, Romania

**Keywords:** neonates, interleukins, umbilical cord blood, early-onset neonatal sepsis, predictor

## Abstract

Neonatal sepsis (NS) is a major cause of morbidity and mortality in both preterm and term infants; early-onset NS (EONS) occurs in newborns within the first 72 h of life. Cytokines are messengers with low molecular weight that are produced by macrophages and lymphocytes in response to antigenic stimulations or products of inflammation. Different interleukins (IL) have higher values in EONS, when detected from peripheral venous blood. This review aims to analyze if the cytokines determined from the umbilical cord blood (UCB) of newborns may help in the rapid and accurate diagnosis of EONS in newborns originating from pregnancies with maternal–fetal infectious risk. Three databases, namely, PubMed, Scopus, and Web of Science, were searched for original research articles that assessed the relationship between interleukins and EONS. The search results retrieved a number of 18 articles that complied with the inclusion and exclusion criteria. Some studies report that neonates with EONS had higher umbilical plasma levels of cytokines such as IL-1ß, IL-6, IL-8, IL-10, IL-18, and IL-27. However, results are controversial, as many authors failed to establish the cut-off values of cytokines detected from UCB that may predict EONS. The main limitations of the current studies remain the small study samples, the heterogeneous population, and the lack of stratification of the studied population according to gestational age (GA). The cytokines that seem to be more accurate in the early diagnosis of EONS, as reported by the majority of the studies, are IL-6 and IL-8. The level of these cytokines may guide clinicians in the careful administration of antibiotics, thus aiding in the overall reduction of antimicrobial resistance.

## 1 Introduction

Neonatal sepsis (NS) is a major cause of morbidity and mortality in both preterm and term infants; early-onset NS (EONS) occurs in newborns within the first 72 h of life (Eichberger and Resch, 2022; Schleier et al., 2023). Transplacental infection or an ascending infection from the cervix may be caused by organisms that colonize the mother’s genitourinary tract and pass through the colonized birth canal during delivery (Abdel-Hakim et al., 2019). The microorganisms most commonly associated with EONS include Group *B Streptococcus* (GBS), *Escherichia coli*, *Coagulase-negative Staphylococcus*, *Haemophilus infuenzae*, and *Listeria monocytogenes* (Abdel-Hakim et al., 2019; Kosmeri et al., 2024).

The diagnosis of EONS may be the greatest and most difficult challenge for a neonatologist (Shahkar et al., 2011). With EONS, the newborn may present a history of feeding intolerance, irritability, excessive sleepiness, or “just not looking right” (Ershad et al., 2019; Stein et al., 2023). Vital sign derangements include both hypothermia and fever; there may be tachycardia or bradycardia, signs of poor perfusion, including cool and pale extremities, and a rapid thready pulse (Ershad et al., 2019). Respiratory symptoms and signs are common in EONS, including grunting, nasal flaring, use of accessory muscles of respiration, cyanosis, and episodes of apnea (Ershad et al., 2019). Neurological symptoms and signs include lethargy, seizures, irregular respiration, high pitched cry, hypotonia, hypoactive deep tendon reflexes, and abnormal primitive reflexes (Ershad et al., 2019). Gastrointestinal signs include decreased feeding, vomiting, diarrhea, jaundice, abdominal distension, and hepatosplenomegaly (Ershad et al., 2019). Skin findings include petechiae, impetigo, cellulitis, and abscess (Ershad et al., 2019). Underlying metabolic acidosis secondary to poor perfusion can manifest as tachypnea and respiratory distress in the absence of respiratory tract infection (Ershad et al., 2019).

The signs and symptoms of EONS are similar to those with non-infectious inflammation. This brings more difficulties in the clinical diagnosis, when the infection source cannot be determined (Xing et al., 2023). The current guidelines contain three general approaches toward identifying newborns with an increased risk of EONS: a categorical risk factor assessment, a multivariate risk assessment (EONS calculator), and a risk assessment primarily based on newborn clinical conditions (Nusman et al., 2023). There are many complications associated with EONS, including septic shock, multiple organ failure, and death (Attia Hussein Mahmoud et al., 2023).

Many biomarkers have been studied to determine their utility in the diagnosis of EONS; the biomarkers are divided into acute-phase proteins, cell surface antigens, cytokines and chemokines, and soluble adhesion molecules (Gude et al., 2022). C-reactive protein (CRP), procalcitonin, serum amyloid A, and hepcidin are acute-phase proteins that are used to diagnose EONS; interleukin-1 (IL-1), IL-6, IL-8, tumor necrosis factor (TNF), and soluble TNF receptor (sTNFR) are cytokines and chemokines implicated in the diagnosis of EONS (Gude et al., 2022).

Cytokines are messengers with low molecular weight that are created by macrophages and lymphocytes in response to antigenic stimulations or products of inflammation (Boskabadi and Zakerihamidi, 2018; Gude et al., 2022). IL-6 is an inflammatory cytokine, produced immediately after induction of inflammation, peaking 6 h afterward; IL-6 measurement results can be obtained within a few hours after umbilical cord blood (UCB) sampling, so clinicians can make immediate treatment decisions (Fadilah et al., 2022). IL-8 is a pro-inflammatory cytokine and is predominantly produced by monocytes, macrophages, and endothelial cells. IL-8 regulates the migration and activation of leukocytes; IL-8 level can be evaluated promptly within 1–3 h of infection, and its half-life is less than 4 h (Zhou et al., 2015).

Unfortunately, the gold-standard diagnostic technique of EONS, the blood culture, requires at least 24–72 h to deliver a reliable result, and the prenatal use of antibiotics further decreases its accuracy (Qiu et al., 2018; Liu et al., 2020). Despite its non-specific clinical symptoms, EONS is highly fatal and can lead to serious long-term complications (Liu et al., 2020; Goyal et al., 2024).

The Kaiser Permanente (KP) group in Northern California applied the Bayesian approach to create a multivariate model of predicting infant-specific EONS risk—the sepsis risk calculator (SRC), derived and validated from a case-control study of blood culture-proven EONS (Puopolo et al., 2011; Escobar et al., 2014; Kuzniewicz et al., 2017; Goel et al., 2020). The SRC derives the “sepsis risk score” (SRS) at birth by modifying the population risk with intrapartum risk factors of the specific mother–infant pair; it then incorporates the infant’s clinical status to compute the final SRS (Goel et al., 2020). Although the SRS computation algorithm is evidence based, the clinical management recommendations (based on the SRS) are a consensus opinion of the KP clinicians (Goel et al., 2020). For example, infants with SRS <1 are assigned normal care, SRS 1–3 receive enhanced observation ± blood culture, and symptomatic or SRS >3 are treated with antibiotics (Goel et al., 2020).

No currently available test is able to provide perfect diagnostic accuracy, and false-negative as well as false-positive results may occur (Cobo et al., 2013; van Leeuwen et al., 2024). Thus, empirical antibiotic therapy remains the current practice in all neonates with suspicion of EONS (Cobo et al., 2013). However, empirical treatment also increases exposure to adverse drug effects and nosocomial complications and poses a high risk of developing resistant strains (Cobo et al., 2013).

Considering that the majority of studies have left a door open for searching new diagnostic tools that will help the clinicians in diagnosing EONS, this review *aims* to analyze if the cytokines determined from the UCB of newborns may help in the rapid and accurate diagnosis of EONS in newborns originating from pregnancies with maternal–fetal infectious risk.

## 2 Materials and methods

We searched the PubMed, Scopus, and Web of Science databases for articles indexed from 1987 through 3 September 2024 that assessed the relationship between interleukins and EONS. We searched for these articles without restrictions on publication date. The search terms used were “interleukin” AND “early” AND “onset” AND “neonatal” AND “sepsis.” We aimed to include human-based studies conducted on neonatal populations, literature data published in English language, and research articles that analyzed a correlation between levels of cytokines determined from UCB and EONS. Exclusion criteria consisted of non-English language literature data, studies that did not meet our article objective, articles without available abstracts, duplicates, case reports, editorials, letters to the editor, review articles and meta-analyses, as well as experimental, animal studies.

The article selection process first consisted of exclusion of duplicates and then of the records written in non-English language or without available abstracts, a task which was performed by authors RMA and SMO. Each of the three authors of the article examined the title and abstracts of the identified reports to exclude irrelevant articles for the reviewer’s objectives. SMO and RMA accessed the full-length text of the manuscripts and checked for compliance to the inclusion criteria. Eventual disagreements between authors had been thoroughly debated and discussed by all authors. The inclusion of each individual record belonging to this review was established upon mutual agreement.

The following information was extracted from articles belonging to the final selection pool: author name, year of publication, type of study, population and study group assignment, and main findings of the article related to IL levels detected from UCB and the association between these ILs and EONS.

## 3 Results

**Figure 1:**
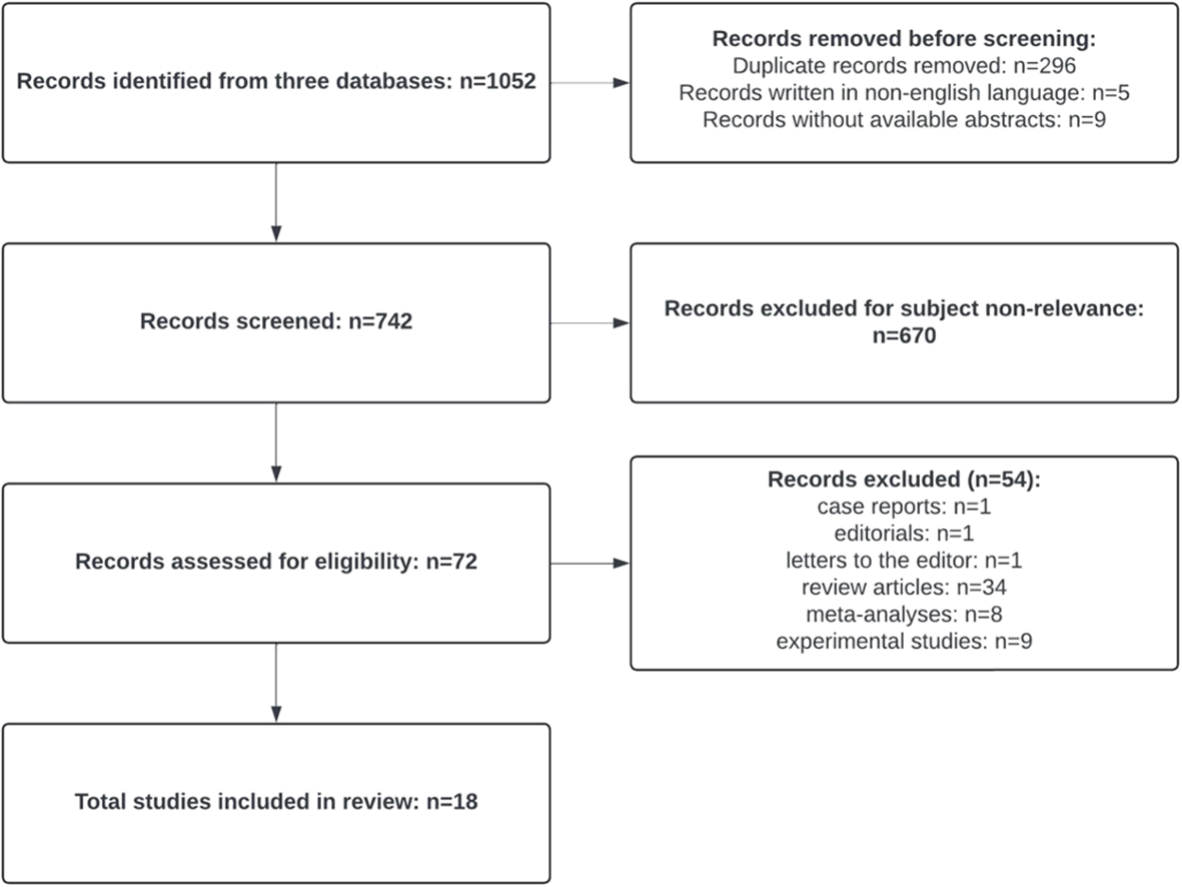
PRISMA flowchart for assessment of eligible studies.

The search retrieved a total number of 1,052 records. After exclusion of 296 duplicate articles, 5 non-English language articles and 9 articles without an available abstract, a number of 742 articles were screened. Then, 670 articles that were not in line with the reviewer’s objectives were excluded (637—because they did not study the level of cytokines in EONS and 33—because they did not evaluate the level of cytokines in UCB). A number of 72 relevant articles were assessed, and after the exclusion of case reports, editorials, letters to the editor, review articles, meta-analyses, and experimental studies, 18 admissible articles were systematically selected. The article selection process is detailed in **Figure 1** and was performed in accordance with the PRISMA 2020 statement (Page et al., 2021).

A summary of the main findings of the studies that complied with our election criteria is provided in **Table 1**.

**Table 1:** Characteristics of selected neonatal studies that assessed the role of the level of UCB cytokines in the diagnosis of EONS.

No. crt.	Reference (author, year)	Type of study	Population and study group assignment	Cytokines detected from umbilical cord blood (UCB)	Main outcome
1.	(González-Andrade et al., 2019)	Epidemiological, observational, analytical, cross-sectional study	200 newborns: - 100 full-term newborns with risk factors for EONS - 100 full-term newborns without risk factors for EONS	IL-6 was determined by conventional electrochemiluminescence immunoassay (ECLIA) method	Correlation of risk factors, such as gestational age (GA) in weeks, weight, prenatal preeclampsia, urinary tract infection (UTI) prior to birth, bacterial vaginitis/vaginosis, and values of IL-6 ≤14 pg/µl
2.	(Døllner et al., 2001)	Case-control study	52 infected neonates: - 33 non-infected but sick controls - 99 healthy controls	Umbilical plasma levels of TNF-alpha (TNF-a), IL-1ß, IL-6, IL-8, sTNFRs (p55 and p75), IL-1 receptor antagonist (IL-1RA) were measured by enzyme-linked immune-sorbent assay (ELISA)	EONS was associated with a prenatal immune response with increased TNF-a, IL-1ß, IL-6, IL-8, p55, p75, and IL-1RA levels in umbilical plasma
3.	(Santana et al., 2001)	Retrospective	31 neonates: - Group I—10 newborns who developed EONS with a positive blood culture; - 11 newborns with non-infectious perinatal diseases; Group III—control group, 10 randomly selected patients, matched for GA	IL-6, IL-8 and soluble receptor of IL-2 (sIL-2R) levels were measured using ECLIA IL-1b and TNF-a measurements were done manually by ELISA	UCB levels of IL-6 were related to pathological conditions in the perinatal period (infectious and non-infectious), but IL-8 was a good predictor of EONS
4.	(Fadilah et al., 2022)	Observational prospective cohort study	40 neonates who were born to mothers with sepsis risk factors: - 22 neonates with normal IL-6 levels - 18 neonates with elevated IL-6 levels	IL-6 concentration was measured using the ELISA method	Significantly more infants with elevated IL-6 developed EONS than those with normal IL-6 In neonates with maternal risk factors, an IL-6 level of ≥16.4 pg/ml was associated with an increased risk of EONS
5.	(Cernada et al., 2012)	Observational prospective cohort study	128 neonates	IL-6 was determined in serum using a chemiluminescence enzyme immunoassay in solid phase	UCB IL-6 was a better predictor to initiate treatment in neonates with prenatal infectious risk factors immediately after birth
6.	(Basu et al., 2012)	Observational prospective cohort study	The study group comprised of symptomatic neonates with positive blood culture (n = 36) An equal number of GA-matched asymptomatic neonates without risk factors of sepsis served as controls	IL-6 level was determined by Human IL-6 ELISA kit	UCB IL-6 can be used as a highly sensitive and specific early diagnostic marker of EONS at a cut-off concentration of 40.5 pg/ml
7.	(Berner et al., 2000)	Prospective study	99 neonates: - Septic group A: 9 newborns Non-septic group B: 22 newborns - Control group C: 68 newborns	UCB samples of neonates with EONS were analyzed qualitatively and semiquantitatively by reverse transcriptase polymerase chain reaction (RT-PCR) for messenger RNA (mRNA) expression of TNF-a, IL-1b, IL-6, IL-8, as well as for IL-8 cord plasma levels. Plasma levels were measured by a double sandwich ELISA using a commercial kit specific for human IL-8	The presence of IL-6 and TNF-a gene expression was observed more frequently in septic than in non-septic neonates When using a semiquantitative approach for analyzing IL-8, mRNA levels, high sensitivity (86%) and specificity (96%) were detected when sepsis was achieved
8.	(Perenyi et al., 1999)	Prospective study	22 neonates: - Group I: 8 mothers with chorioamnionitis and their 8 infants having risk factors for infection Group II: 5 mothers and their 9 infants (one set of triplets and one of twins). The mothers did not have chorioamnionitis (Prematurity was the only risk factor for infection) - Group III, the controls: 5 term neonates with no risk factor for infection	IL-6 levels were measured using ELISA method	While UCB of infants born to mothers with no chorioamnionitis had IL-6 levels lower than those of infected mothers, it was questionable that the sensitivity and specificity of blood IL-6 sufficed as an index of EONS and justified its use for this purpose
9.	(El Farargy et al., 2018)	Prospective case-control study	85 neonates divided into 3 groups: - Group 1 (Control): 10 cases in which UCB levels of IL-6 and IL-8 were normal at birth and CRP was negative at birth and still negative after 3 days - Group 2 (Suspected infection): 45 cases in which UCB level of IL-6 and IL-8 were elevated at birth, and CRP was negative at birth, then became positive after 3 days - Group 3 (Infected): 30 cases in which UCB levels of IL-6 and IL-8 were elevated at birth, and CRP was positive at birth and still positive after 3 days	Serum levels of IL-6 and IL-8 were detected by ELISA	IL-6 and IL-8 were more sensitive than CRP in early prediction of EONS. IL-6 and IL-8 levels should be done for early detection of EONS, which will lead to early diagnosis of EONS with early treatment and subsequent better prognosis
10.	(Najati et al., 2009)	Case-control study	100 low birth weight (LBW) neonates: - Case group: 50 LBW neonates born from mothers with premature rupture of membranes (PROM) - Control group: 50 LBW neonates born from mothers without PROM	IL-8 levels were measured using ELISA method	Increased levels of IL-8 can be used as indicator for early diagnosis of EONS
11.	(Lehrnbecher et al., 1996)	Case-control study	46 neonates: - Septic group: 13 neonates - Non-septic group: 33 neonates	Commercially available enzyme immunoassays were used for determination of IL-6 and IL-8	No correlation between fetal and maternal blood concentrations of IL-6 and IL-8 was observed when the septic and non-septic neonates were compared with their infected and non-infected mothers
12.	(Hatzidaki et al., 2005)	Case-control study	109 neonates: - The PROM group: 58 neonates who were born after PROM The control group: 51 neonates	The level of IL-6 was measured blindly employ-ying ELISA	IL-6 in UCB was the most significant variable for predicting EONS in preterm neonates. IL-6 in maternal blood was indicative of intrauterine environmental threats and might be used to identify pregnancies where intervention would be appropriate
13.	(Satar et al., 2008)	Case-control study	83 mothers and their neonates: - The PPROM group: 42 women with 42 premature neonates (GA 29–35 weeks) with prolonged PROM (PPROM) exceeding 24 h - The control group: 41 women with 41 premature neonates without PPROM	Both mothers’ and cord blood serum were tested for IL-6, IL-8, and TNF-a levels using a microELISA method	IL-8 levels in UCB increased significantly in PPROM and were found to be even higher in patients who later developed necrotizing enterocolitis (NEC). IL-8 and IL-6 levels in mothers’ serum were both elevated in the PPROM group, but TNF-a levels did not differ
14.	(Mercer et al., 2012)	Prospective multicenter randomized trial	614 non-laboring gravidas with PROM were randomized to antibiotic therapy or a matching placebo regimen during conservative management - 222 maternal samples obtained at randomization - 121 maternal samples obtained at delivery - 196 UCB samples - 82 patients had all three samples collected	IL-6, IL-10, granulocyte colony-stimulating factor (G-CSF), TNF-a, and circulating intercellular adhesion molecule-1 (ICAM-1) levels were determined from maternal blood at the time of delivery, and from UCB at the time of delivery	UCB cytokine values were higher than maternal levels suggesting significant fetal/placental contribution. Maternal and UCB cytokine levels were not adequately predictive to be used clinically
15.	(Weeks et al., 1997)	Case-control study	133 preterm newborns: - Affected group: 16 neonates with adverse outcomes: pneumonia (n = 4), NEC (n = 5), sepsis (n = 51), or degrees II–IV intraventricular hemorrhage (IVH) (n = 2) - Unaffected group: 117 neonates	IL-6 levels were measured using ELISA method	UCB IL-6 levels were elevated in neonates who subsequently developed sepsis, congenital pneumonia, NEC, or degrees II–IV IVH
16.	(Cancelier et al., 2009)	Prospective case-control study	120 consecutive preterm neonates with at least one risk factor for EONS: - Sepsis group: 40 newborns (20 cases of proven and 20 cases of highly probable NS) - Controls: 80 newborns	Commercial ELISA kits were used for the detection of IL-6 and IL-10	Cord blood IL-6, IL-10, and oxidative stress markers were significantly higher in infants with NS, and only thiobarbituric acid-reactive substance (TBARS) levels were independently related to the development of NS
17.	(Janec et al., 2023)	Prospective case-control study	20 neonates: Study group (sepsis group): 8 newborns with GA ≤32 weeks, with culture-positive EONS - Control group: 12 newborns without EONS	Soluble markers of inflammation were measured using Luminex-based multiplex assay	In EONS, IL-18 and VEGF-A (vascular endothelial growth factor-A) might be considered part of the diagnostic workup
18.	(Sultan et al., 2023)	Observational analytic study	124 neonates: - Infection group: 48 neonates with both suspected and confirmed EONS - Non-infection group: 76 neonates	The concentrations of IL-27 were measured by sandwich ELISA assay	IL-27 level in the UCB may represent a significant predictive tool for EONS, either alone or in combination with PCT

CRP, C reactive protein; EONS, early-onset neonatal sepsis; ECLIA, electrochemiluminescence immunoassay; ELISA, enzyme-linked immune-sorbent assay; GA, gestational age; G-CSF, granulocyte colony-stimulating factor; ICAM-1, circulating intercellular adhesion molecule-1; IL, interleukin; IL-1RA, IL-1 receptor antagonist; IVH, intraventricular hemorrhage; LBW, low birth weight; mRNA, messenger ribonucleic acid; NEC, necrotizing enterocolitis; NS, neonatal sepsis; PPROM, prolonged premature rupture of membranes; PROM, premature rupture of membranes; RT-PCR, reverse transcriptase polymerase chain reaction; sIL-2R, soluble receptor of IL-2; sTNFR, soluble TNF receptor; TBARS, thiobarbituric acid reactive substances; TNF-a, tumor necrosis factor-alpha; UCB, umbilical cord blood; UTI, urinary tract infection; VEGF-A, vascular endothelial growth factor-A.

### 3.1 IL-6 detected from UCB and EONS

IL-6 levels in UCB was associated with EONS, and it could predict EONS (González-Andrade et al., 2019; Fadilah et al., 2022). González-Andrade et al. showed that in neonates with LBW, who were related to prenatal preeclampsia, prenatal UTI, and vaginitis/vaginosis, IL-6 reached higher values of up to 414 pg/µl, with a 90th percentile of up to 215 pg/µl, which is attributed to the presence of EONS (González-Andrade et al., 2019). The authors considered that IL-6 was a predictor of EONS associated with prenatal risk factors (González-Andrade et al., 2019). Fadilah et al. found elevated IL-6 in 18 subjects, 10 of whom had EONS (Fadilah et al., 2022). Still, out of the 22 subjects with normal IL-6, 3 had EONS (Fadilah et al., 2022). Blood cultures were performed in subjects with clinical EONS, but none of them were positive (Fadilah et al., 2022). Fadilah et al. demonstrated that neonates with elevated IL-6 levels were 5.54 times more likely to develop EONS than those with normal IL-6 levels [RR 5.54 (95% CI 1.68 to 18.25); p = 0.016] (Fadilah et al., 2022). IL-6 level in UCB was significantly associated with clinical EONS and can be used as a quick, early test to predict clinical EONS (Fadilah et al., 2022). Other studies tried to identify cut-off values for IL-6 for accurate prediction of EONS. Weeks et al. discovered that the median IL-6 level in the unaffected group—infants without neonatal complications—was 0 pg/ml (range 0–2,024), and the median IL-6 level in the affected group was 145 pg/ml (range 0–2,628); this difference was statistically significant (p = 0.002) (Weeks et al., 1997).

When the UCB levels of IL-6 were compared with other markers, such as CRP, the authors found that IL-6 levels were better predictors of EONS (Cernada et al., 2012). Cernada et al. showed that IL-6 had superior sensitivity (90% vs. 60%), specificity (87.4% vs. 79%), and positive (37.5% vs. 19.4%), and negative (99% vs. 99.5%) predictive values compared with CRP (Cernada et al., 2012). Basu et al. only included those cases of EONS with positive blood culture to avoid any problems related to the accurate diagnosis; most neonates from their study were preterm and had LBW (Basu et al., 2012). The authors reported that elevated UCB IL-6 concentrations were a better diagnostic tool compared to conventional EONS screening in neonates who subsequently developed EONS with positive blood cultures (Basu et al., 2012).

There was a positive correlation between maternal and UCB IL-6 values. But there were no differences in maternal blood IL-6, even if the mothers had been diagnosed with infections or not (Perenyi et al., 1999). Perenyi et al. found that UCB IL-6 levels in infants of mothers considered to be noninfected were lower than those born to women with chorioamnionitis (Perenyi et al., 1999). Hatzidaki et al. found that IL-6 concentrations in maternal blood, UCB, and neonatal blood were significantly higher in neonates with EONS compared with those without EONS (p < 0.001) with a cut-off concentration of IL-6 in UCB of 108.5 pg/ml for EONS (sensitivity 95%, specificity 100%, positive predictive value 100%, and negative predictive value 97.4%) and cut-off concentration of IL-6 in maternal blood of 81 pg/ml (sensitivity 90%, specificity 97.4%, positive predictive value 94.7%, and negative predictive value 94.9%) (Hatzidaki et al., 2005).

### 3.2 IL-8 detected from UCB and EONS

The UCB level of IL-8 can be used in monitoring high-risk neonates and for early recognition of EONS (Najati et al., 2009). Najati et al. revealed a mean IL-8 level in a study group of 128.12 pg/ml, whereas the mean IL-8 level in the control group was 39.02 pg/ml; there was no positive blood culture and no bacteria isolated (Najati et al., 2009).

When a comparison of the study groups (infected vs. non-infected) was differentially made in accordance with GA, in the group of premature newborns, the median IL-8 value was 248.9 pg/ml (normal range <31.5 pg/ml), and in the group of term newborns, the median IL-8 value was 30.9 pg/ml (normal range <31.5 pg/ml); these results suggested that assessments of IL-8 in the umbilical plasma may identify EONS accurately, but only among preterm neonates (Døllner et al., 2001). Significantly elevated values (p < 0.0003) were shown in cases with PROM compared to cases without PROM (medians 67.5 vs. 29.5 pg/ml), which highlighted the strong relationship between IL-8 elevation and PROM (Najati et al., 2009). Satar et al. also showed that the mean IL-8 levels in UCB and mothers’ serum were significantly higher in the PPROM group (p < 0.001, p< 0.005) (Satar et al., 2008). Neonates who developed NEC had higher IL-8 levels in their UCB when compared to those without NEC (p < 0.05), and PPROM increases the risk of NEC. So, newborns with PPROM and higher IL-8 levels in UCB might be considered at possible risk of NEC (Satar et al., 2008).

Santana et al. tried to investigate whether UCB levels of CRP, IL-1beta, IL-6, IL-8, TNF-a, and SIL-2R were useful markers in the diagnosis of EONS. For this purpose, UCB samples were obtained at birth from 261 neonates, but 5 of these newborns were excluded from the study. The newborns were divided into three groups: Group I—contained 10 newborns that developed positive blood culture EONS, Group II—included 11 newborns with non-infectious perinatal diseases, and Group III—served as the control group and included 10 neonates randomly selected, matched for GA, from the total of 235 healthy newborns included in the study from the beginning. IL-8 was significantly elevated in Group I (389.3 ± 115.9 pg/ml) compared with Group II (30.2 ± 5.1 pg/ml) (p < 0.05) and Group III neonates (33.9 ± 8.6 pg/ml) (p < 0.05). IL-8 was a good predictor of EONS (Santana et al., 2001).

### 3.3 Combination of IL-6 and IL-8 detected from UCB and EONS

UCB measurements of IL-6 and IL-8 were useful in identifying neonates with EONS (Lehrnbecher et al., 1996; El Farargy et al., 2018). El Farargy et al. showed that the levels of IL-6 and IL-8 were significantly higher in suspected, infected neonate group than in the control group (El Farargy et al., 2018). IL-6 and IL-8 were more sensitive than CRP in the early prediction of EONS. Therefore, the study suggests that IL-6 in combination with IL-8 levels should be routinely measured for early detection of EONS. This will lead to early diagnosis of EONS, with early treatment and subsequent better prognosis (El Farargy et al., 2018).

Lehrnbecher et al. stated that measured concentrations of IL-6 and IL-8 in fetal and maternal blood did not correlate indicating that the neonate’s response to EONS is clearly different from that of the mother’s (Lehrnbecher et al., 1996). UCB combination of measurements of IL-6 and IL-8 were useful in identifying neonates with EONS (Lehrnbecher et al., 1996).

Neonates with EONS had significantly higher levels of IL-6 and IL-8 than healthy infants (Santana et al., 2001). The results suggested that the levels of IL-8 in UCB had diagnostic value in the identification of newborns that will develop EONS (Santana et al., 2001). Conversely, the value of IL-6 in identifying these infants was limited because it increased in the UCB of neonates with infectious as well as non-infectious conditions; this highlighted the higher ability of IL-8 compared to IL-6 in the prediction of EONS (Santana et al., 2001). Levels of IL-8 in UCB higher than 112 pg/ml were useful for the identification of newborns that were likely to develop EONS. IL-6 did not appear to be useful in the differentiation of neonates with infections and those with non-infectious pathological conditions (Santana et al., 2001).

### 3.4 Combination of pro-inflammatory and anti-inflammatory ILs and EONS

Studies have shown that in pathways of inflammation, the pro-inflammatory cytokines are represented by IL-1ß, IL-1α, IL-6, IL-8, IL-18, and TNF-a; the anti-inflammatory cytokines are represented by IL-10, IL-1R, and IL-27 (Fahey and Doyle, 2019; Tapia et al., 2019; Seman et al., 2020; Al-Qahtani et al., 2024).

Assessments of IL-1ß, IL-6, and IL-8 in the umbilical plasma may identify EONS accurately, but only among preterm neonates (Døllner et al., 2001). Døllner et al. found that neonates with EONS had higher umbilical plasma levels of TNF-a, IL-1ß, IL-6, IL-8, p55, p75, and IL-1RA than in healthy neonates; that was sustained by an extensive cytokine response established prenatally in relation to EONS (Døllner et al., 2001). Term infected neonates had moderately elevated mediator levels in the umbilical plasma, but among term neonates, none of the mediators differed significantly between infected and non-infected sick controls—newborns with clinical symptoms (Døllner et al., 2001).

Santana et al. postulated that the levels of IL-8 in UCB were of diagnostic value in the identification of newborns that will develop EONS at levels higher than 112 pg/ml (Santana et al., 2001). IL-6 increased in the UCB of neonates with infectious as well as non-infectious conditions (Santana et al., 2001). The remaining cytokines in UCB were not useful for EONS prediction (Santana et al., 2001).

Cord plasma levels of IL-8 were significantly elevated in septic infants, when compared to infants with unconfirmed EONS and with healthy infants (Berner et al., 2000). Satar et al. reported that mean IL-8 levels in cord blood and mothers’ serum were significantly higher in the PPROM group (p < 0.001, p< 0.005); IL-6 levels found in mothers’ serum were significantly higher than those found in the control group (p < 0.01), but levels of IL-6 in UCB were similar (p > 0.05); TNF-a levels were also similar in both groups (p > 0.05) (Satar et al., 2008).

Mercer et al. revealed that UCB levels were higher than maternal levels obtained at randomization and at delivery for IL-6 and for G-CSF. Maternal ICAM-1 levels from blood obtained at randomization and at delivery were higher than those obtained from UCB; IL-10 levels were similar between UCB [10 (7–20) pg/ml], maternal randomization [3 (3–5) pg/ml], and maternal delivery [27 (16–62) pg/ml] samples. TNF-a levels were rarely positive, with over 99%, 95%, and 90% of the samples having undetectable levels at randomization, delivery, and in UCB (Mercer et al., 2012).

UCB levels of IL-6, IL-10, TBAR, and protein carbonyl were higher in the sepsis group (grouping proven and highly probable EONS) when compared with the control; only TBARS and IL-6 were higher in the proven sepsis group in comparison with highly probable septic newborns; TBARS was a better predictor for the occurrence of EONS in this study (OR for TBARS was 2.16 vs. OR for IL6 was 1.2) (Cancelier et al., 2009).

Regarding the neonatal values of cytokines, Mercer et al. showed that IL-6 and IL-10 were higher in infants developing confirmed EONS (p ≤ 0.04); G-CSF, IL-6, and IL-10 levels were significantly associated with EONS before discharge (p ≤ 0.03); TNF-a and IL-10 levels in UCB were not significantly increased in relation to any adverse outcomes; no UCB marker was significantly associated with neonatal pneumonia (Mercer et al., 2012).

Two new cytokines were studied recently: IL-18 and IL-27. Janec et al. highlighted a significant increase in IL-18 in the UCB of the sepsis group when compared to controls (mean ± SEM, 104.7 ± 30.4 pg/ml vs. 52.7 ± 5.6 pg/ml, p = 0.02); the VEGF-A (vascular endothelial growth factor-A) level in the UCB was not affected by EONS (sepsis group: 32.0 ± 6.4 vs. control group: 40.5 ± 23.1 pg/ml, p = 0.77); all other measured markers did not show significant differences between the sepsis and control groups, including endocan, angiopoietin 2, IL-1α, soluble mucosal addressin cell adhesion molecule (sMadCAM), soluble vascular cell adhesion molecule 1 (sVCAM-1), IL-6, TNF, and soluble VEGF receptors 1 and 2 (sVEGF-R1, sVEGF-R2) (Janec et al., 2023). Significantly higher levels of IL-27 were found in UCB of newborns from the infection group (p < 0.01), and IL-27 continued to show significantly higher levels in the infection group compared to those in the non-infection group (p < 0.01) at 24 h after birth (Sultan et al., 2023).

## 4 Discussions

EONS resulting from bacterial bloodstream infections remains a serious clinical concern and is currently defined as a “life-threatening organ dysfunction caused by a dysregulated immune response that occurs as the result of an infection” (Shankar-Hari et al., 2016; Greenfield et al., 2021). The dysregulated immune response is obvious through the initial hyperinflammatory response driven by proinflammatory cytokines and chemokines (Shankar-Hari et al., 2016; Greenfield et al., 2021). Campos et al. argued that routine cytokine assays entail increased cost, and they have not reliably established reference ranges, as cytokine levels vary widely from person to person and are highly influenced by external factors (Campos et al., 2010).

Cytokines may have great potential for diagnosis of EONS and have been studied for many years. The promising determination of cytokines from UCB in cases of maternal–fetal infectious risk remains a challenge because of the variety of the study populations enrolled, which could have led to such different results between studies. IL-6 seems to be the cytokine that was mostly studied and also IL-6 may be a good predictor of EONS. This statement was sustained by the studies of González-Andrade et al. and Basu et al. that described UCB IL-6 concentrations as a highly sensitive and specific early diagnostic parameter of EONS (Basu et al., 2012; González-Andrade et al., 2019). However, the study population of González-Andrade et al. was represented by full-term newborns, while that of Basu et al. was represented by preterm, LBW neonates (Basu et al., 2012; González-Andrade et al., 2019). Available conventional sepsis screening markers, including hematologic indices and CRP, were not reliable early markers for EONS (Basu et al., 2012).

IL-6 concentrations in UCB, and to a lesser degree in maternal blood, that were taken during delivery were very sensitive, reliable, and early markers of EONS. They could provide an accurate indication of whether a neonate will develop EONS, thus, offering the opportunity for prompt diagnostic and aggressive therapeutic intervention (Hatzidaki et al., 2005). In contrast to this study, Mercer et al. stated that initial maternal plasma markers were not associated with adverse neonatal outcomes; maternal plasma markers evaluated at delivery are less closely associated with neonatal complications than UCB markers (Mercer et al., 2012). UCB reflected the intrauterine fetal environment and was associated with subsequent infectious and composite morbidity (Mercer et al., 2012). According to the authors, the combination of levels of multiple markers and clinical findings could further refine the predictive value of these tests, but their sample size was inadequate to evaluate this (Mercer et al., 2012). The higher levels of IL-6 and G-CSF seen in UCB compared to maternal blood suggested strong fetal/placental participation in the inflammatory response related to intrauterine infection and preterm parturition; these elevations reflected a subclinical inflammatory process that was present at the time of initial evaluation of these women (Mercer et al., 2012).

An increase in IL-8 in UCB might indicate fetal inflammation, but no significant increase was seen in regard to EONS in the newborns with PPROM, when compared to the control group (p > 0.05) (Satar et al., 2008).

Cernada et al. speculated that performing analytical assays in UCB could avoid repeated blood extractions in the first hours of postnatal life. The values of IL-6 can be ready in 30–60 min as part of the routine clinical laboratory, so it could be useful to take decisions in the first hours of life depending on these biomarkers (Cernada et al., 2012). A limitation of their study was the fact that the authors enrolled preterm (50% of neonates were preterm) and full-term newborns that had completely different immune responses to EONS (Cernada et al., 2012). Similar to Cernada et al., Lehrnbecher et al. concluded that IL-6 and IL-8 may indicate EONS earlier than CRP (Lehrnbecher et al., 1996).

Excessive inflammation in EONS might be involved in the damage associated with EONS (Chen et al., 2022). Several studies have demonstrated that serum pro-inflammatory TNF-a, IL-1β, IL-6, and IL-8 levels are rapidly and strikingly elevated in EONS (Kurt et al., 2007; Wu et al., 2016; Leal et al., 2019). In contrast to these studies, Perenyi et al. concluded that elevated UCB levels may predict maternal chorioamnionitis, rather than the EONS (Perenyi et al., 1999). IL-6 becomes detectable within 1 h after triggering an infectious stimulus, reaches its plateau in 4 to 6 h, and the IL-6 release may be essentially abolished by 36 h, even while the infection remains unchallenged; as IL-6 blood level drops, acute phase reactants (such as CRP) increase (Perenyi et al., 1999).

Septic neonates had significantly higher UCB levels of IL-6 and IL-8 than non-septic neonates. But determinations of IL-6 in neonatal peripheral blood and from UCB showed comparable results in indicating EONS (Lehrnbecher et al., 1996). The association of IL-6 and IL-8 detected from UCB with EONS was also sustained by El Farargy et al. But in their study, EONS was more common in preterm than in full-term neonates (p = 0.007) (El Farargy et al., 2018).

Weeks et al. speculated that UCB IL-6 levels could assist neonatologists in determining which infants were at greatest risk for infectious morbidity, NEC, and high-grade IVH. The authors hypothesized that UCB IL-6 levels could be useful in directing the use of diagnostic tests and determining which neonates require postnatal therapies, such as antibiotics (Weeks et al., 1997). Neonates with EONS had higher levels of inflammatory and oxidative stress markers in UCB before the onset of symptoms; UCB IL-6, IL-10, and oxidative stress markers were significantly higher in infants with EONS, even in the proven sepsis group (Cancelier et al., 2009).

Recent studies have brought the following new markers to the scene: IL-18 and IL-27. A significant increase in IL-18 in the UCB was observed in the sepsis group, but all the other markers that were investigated did not show significant differences (Janec et al., 2023). IL-27 had a better performance in distinguishing neonates with true infection from neonates without infection, and a combined performance of IL-27 with PCT in the UCB and at 24 h of life showed greater prediction of EONS with p < 0.01 for each parameter/parameter combination (Sultan et al., 2023). IL-27 had an increased risk of EONS with an odds ratio of 9.13 and p < 0.01 (Sultan et al., 2023).

Nakstad et al. stimulated UCB samples from 20 healthy term pregnancies for 2 h with a GBS III isolate from a patient and a commercially available GBS Ia strain; nonstimulated samples served as controls (Nakstad et al., 2016). The authors concluded that IL-6, IL-8, and potentially CD11b could be useful in diagnosing neonatal GBS infection in an early stage (Nakstad et al., 2016). Only in the study of Berner et al. were the levels of UCB IL-8 more elevated after cesarean section (114.9 vs. 23.1 pg/ml, p < 0.001) (Berner et al., 2000).

Neither serum IL-6 nor IL-10 alone was a useful diagnostic index of EONS in equine neonates, but the number of animals involved in the study was too small (15 septic neonatal foals vs. 15 age-matched control foals) for the identification of a concrete value; the serum IL-6:IL-10 ratio was likely to provide a valuable prognosticator for EONS (Burton et al., 2009).

Intrapartum antibiotic exposure can result in a partially treated infant delaying the onset of clinical signs and symptoms of infection and further complicating the expedient definitive diagnosis of EONS (Najati et al., 2009). This information was in opposition to that of Mercer et al. who claimed that evaluated maternal systemic inflammatory markers were marginally affected by antibiotic treatment during conservative management of PROM remote from term. That evaluation of these systemic inflammatory markers at initiation of treatment was not likely to provide adequately predictive information regarding subsequent adverse maternal and neonatal outcomes. While increased levels of the evaluated UCB pro-inflammatory cytokines were associated with subsequent adverse neonatal outcomes, significant overlap in values between newborns with and without subsequent complications limited the predictive and clinical value of these markers for use in clinical practice; the authors concluded that it is plausible that the inflammatory cascade cannot be stopped once initiated (Mercer et al., 2012).

Unfortunately, there is no adequate laboratory test, single or in combination, which has sufficient sensitivity and specificity to allow neonatologists to safely rule out EONS. For that reason, all newborns with clinical signs that are suggestive of EONS should be treated empirically with antibiotics, after peripheral and blood cultures have been taken. Careful observation of apparently good-looking full-term newborns that are born from pregnancies with infectious risk is fundamental during the first 24 h of life. The main limitations of current studies remain the small study samples, the heterogeneous study populations included, and the lack of stratification of the studied population according to GA. GA groups have different risk factors that may be associated with EONS. The results might have differed if the GA-dependent study group division would have been considered as follows: full-term neonates—more than 37 weeks of gestation, preterm neonates with GA less than 24 weeks of gestation, 24–28 weeks of gestation, 28–32 weeks of gestation, 32–34 weeks of gestation, and 34–37 weeks of gestation. Another heterogeneity of the studies was caused by different laboratory techniques used to quantify cytokine levels. Future studies should focus on establishing standardized cut-off values for IL-6 and IL-8 accounting for variations in GA and maternal risk factors.

Practical applications of the findings are huge because the collection of blood from UCB at birth is minimally invasive for the newborn. Cytokines detected from UCB may predict from birth which newborns are at risk of presenting EONS. However, we need precise cut-off values of IL-6 and IL-8 to use them in clinical practice. UCB levels of IL-6 and IL-8 may be cost effective, and this approach of predicting EONS may reduce unnecessary antibiotic use.

## 5 Conclusions

It is well known that EONS contributes to morbidity and mortality in the neonatal period, so that early diagnosis and early and targeted treatment may improve its prognosis and outcome. Testing blood from the UCB is less invasive. The cytokines that seem to be more accurate in early diagnosis of EONS in many studies are IL-6 and IL-8. IL-6 and IL-8 levels have been shown to be rapidly released in response to infection often before the onset of clinical symptoms and before routine positive laboratory tests. The incidence of EONS varies in countries around the world. There is a need for further multicenter, large-sized studies, such as prospective cohort studies that will assess the role of IL-6 and IL-8 detected from both maternal blood and UCB in early diagnosis of EONS and in reducing the abuse of antibiotics. The methodology needs to take account of GA stratification. GA-dependent references for each marker are also claimed. Moreover, validated cut-off values of these cytokines may guide clinicians in the careful administration of antibiotics which will overall reduce antimicrobial resistance. The potential integration of cytokine biomarkers with machine learning models is promising and should also be considered in further studies.
